# Labour Pain: A Comprehensive Review of Perceptions, Experiences, and Sociocultural Influences on Pain and Its Management Practices

**DOI:** 10.7759/cureus.86540

**Published:** 2025-06-22

**Authors:** Archita Tandon, Kanwal Gujral, Vipin Gupta

**Affiliations:** 1 Department of Anthropology, University of Delhi, New Delhi, IND; 2 Institute of Obstetric and Gynaecology, Sir Ganga Ram Hospital, New Delhi, IND; 3 Institute of Obstetrics and Gynaecology, Sir Ganga Ram Hospital, NEW DELHI, IND

**Keywords:** experience, labour pain, pain management, perceptions, sociocultural factors

## Abstract

Childbirth is a deeply personal and subjective experience for women, shaped by individual expectations and sociocultural perceptions. Many women approach labour with specific mental imagery and expectations regarding pain and the birthing process. However, these expectations often differ from the actual experience, which can result in emotional distress during and after childbirth. This scoping review systematically searched PubMed, Scopus, and Web of Science databases to identify studies published between 1984 and 2024 focusing on labour pain perception and experience. Only peer-reviewed articles in English were considered. This review identified three major themes that highlighted labour pain, pain management, and the sociocultural factors that were associated with it. It has revealed that the experience of women was consistent with what they had perceived about pain, while it was inconsistent in terms of perception of pain relief during labour. However, the sociocultural paradigm did hold its place in meddling between the perceptions and experiences of these women during labour pain and management. The exploration of personal attributes and sociocultural dynamics that shaped the women’s ascription of meanings to labour pain add context to how a similar physiological experience can be perceived and experienced differently.

## Introduction and background

Labour pain is a unique, multidimensional phenomenon that differs from typical pain associated with injury or illness. It is a physiological part of childbirth that intensifies as labour progresses [1-4]. Labour is a natural physiological process marked by rhythmic and progressively intense uterine contractions. These contractions lead to cervical effacement and dilation, alongside the stretching of the vaginal canal and perineum, and pressure on the surrounding pelvic structures, all of which contribute to the pain experienced during childbirth [5,6]. Among the various forms of pain encountered by women during their reproductive years, labour pain is often considered the most intense and overwhelming [7]. Childbirth is a distinctly female experience, shaped by gender socialisation and the perceptions women hold as ‘pregnant women’ in their communities. These perceptions are influenced not only by the physiological sensations of pain but also by culturally embedded norms and shared community narratives about what childbirth should entail. For instance, in a study by Callister et al. (2000), women from Jordan described labour pain as a test of maternal endurance and virtue, leading many to refuse pain relief to align with culturally valorised ideals of strength and self-sacrifice during childbirth [8]. This demonstrates how cultural expectations can shape not only women's interpretations of pain but also their actual decisions and behaviours during labour. Studies have shown that women often balance between idealised perceptions and real experiences of labour pain, which can cause distress when expectations are unmet [9,10]. The way governments facilitate childbirth, such as the Janani Suraksha Yojana in India, a conditional cash transfer program in India, homebirth policies in Sweden, or lead maternity care models (continuity of care provided by a primary midwife) in New Zealand, influences the childbirth experience. The sociocultural context plays a significant role in shaping women’s sense of autonomy during labour [3,10-13].

A systematic review on women’s expectations and experience of pain relief highlights the gap between expectations and reality, as well as women’s involvement in decision-making [14], but it does not address how sociocultural factors like ethnicity, religion, and socioeconomic status shape perceptions and experiences of labour pain. A 2019 narrative review focused on the immediate environment of women during labour, such as caregivers’ influence [15], whereas the present scoping review explicitly explores the broader context of women’s decisions, perceptions, and experiences of labour pain and management. Existing reviews primarily focus on pain interventions [16,17] or pain assessment tools [18]. This scoping review aims to examine women’s perceptions of labour pain and its management, with particular emphasis on sociocultural factors including ethnicity, religion, socioeconomic status, and community norms that influence these perceptions and experiences.

Labour pain is not solely a biological event; it is deeply embedded within sociocultural frameworks that shape women's expectations, perceptions, and coping mechanisms. Cultural narratives, familial norms, religious beliefs, and state-supported maternity policies all influence how women make sense of their pain and choose whether to accept, endure, or seek relief from it. Understanding these layers is essential, as previous studies have not comprehensively addressed the interplay between physiological pain and the sociocultural environment [19]. This review therefore adopts a scoping methodology to map the breadth of interdisciplinary literature, identify thematic trends, and explore how women ascribe meaning to labour pain through cognitive and social lenses. By doing so, it seeks to explain why some women cope well while others report significant distress and how sociocultural determinants mediate these differing pain experiences.

## Review

Methodology

This review was conducted in accordance with the Preferred Reporting Items for Systematic Reviews and Meta-Analyses Extension for Scoping Reviews (PRISMA-ScR) guidelines. A comprehensive literature search was performed across three databases - PubMed, Web of Science, and Google Scholar - to identify relevant studies published between January 1, 1984 and December 28, 2024. The search strategy used a combination of terms, including "labour pain" OR "labor pain", AND "perception", "experience", "pain management", "analgesia", "socio-cultural factors", and "cultural influences" in both single and combined formats. Google Scholar was included to capture grey literature, although its limitations in transparency, replicability, and specificity are acknowledged. The first hundred results sorted on the basis of relevance by Google Scholar were considered for this review.

A narrative synthesis approach was employed to integrate findings across diverse study designs. Thematic content analysis was conducted using a hybrid coding framework. Initially, a deductive approach guided by the study’s research questions and key conceptual domains (such as perception of pain, pain management strategies, and sociocultural influences) was used to structure preliminary categories. As the review progressed, an inductive process allowed for the emergence of new codes and subthemes directly from the data, ensuring that context-specific and unanticipated insights were captured. Full-text articles were reviewed and coded independently by two reviewers, with thematic convergence reached through iterative discussion. These excerpts were then systematically coded, and codes were grouped into broader themes through iterative comparison and clustering. This process resulted in the identification of three primary themes: (1) the nature and intensity of labour pain, (2) women's responses to pain relief interventions, and (3) sociocultural factors shaping pain experience. Coding and thematic development were conducted by two reviewers independently and reconciled through discussion to ensure consistency and reduce interpretive bias.

We included studies that explored women's experiences and perceptions of labour pain in both institutional and non-institutional settings, and those that discussed pharmacological and non-pharmacological pain management approaches. We considered quantitative, qualitative, and mixed-method studies, including randomised controlled trials (RCTs), observational studies, cohort studies, cross-sectional studies, and case reports where the perception or experience of labour pain was discussed. The inclusion of case reports was justified as they provide unique insights into culturally specific pain perceptions that are often underreported. In addition, quality appraisal or risk of bias assessment was not performed, consistent with standard scoping review methodology.

We excluded articles that focused primarily on caregiver or clinician perspectives, specific physiological pain types unrelated to labour, autobiographical accounts, and theoretical commentaries unless they presented novel conceptual frameworks directly relevant to labour pain experience.

The articles were independently searched by A.T. and N.K. The initial screening to exclude duplicates and articles without available full text was also performed by A.T. Titles and abstracts were reviewed by both A.T. and N.K. Disagreements during full-text review were resolved by consensus, or by adjudication from two additional authors (V.G. and K.G).

Results

Study Selection

**Figure 1:**
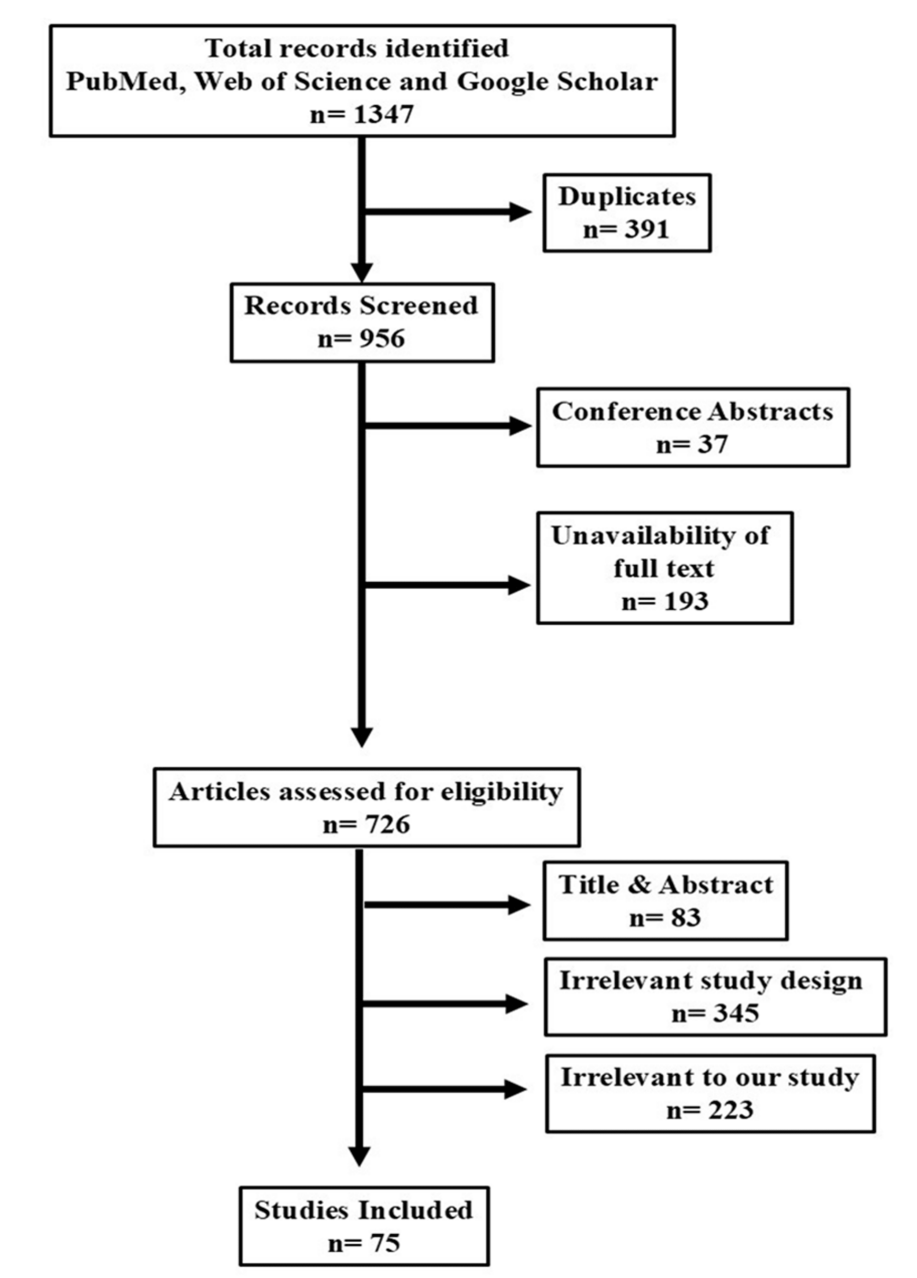
Preferred Reporting Items for Systematic Reviews and Meta-Analyses Extension for Scoping Reviews (PRISMA-ScR) diagram of the included articles

The initial database search identified 1,347 results. After removing duplicates, 956 studies remained, as shown in Figure 1 (PRISMA-ScR flow diagram). Then, 230 studies were excluded due to being conference abstracts (n = 37) or lacking full text (n = 193). Another 651 studies were excluded after screening based on the title/abstract (n = 83), study design (n = 345), or relevance (n = 223). A total of 75 studies met the inclusion criteria. These 75 studies reflected a rich diversity in methodology and context. They included qualitative explorations, cross-sectional surveys, observational studies, randomised trials, and a few case reports - each contributing unique insights into women’s experiences of labour pain. Geographically, the literature drew from a wide range of settings, with many studies conducted in Nigeria, Ghana, India, and Jordan, as well as high-income contexts like Sweden, Australia, the United Kingdom, and New Zealand. The studies captured experiences from both institutional settings, such as hospitals and maternity wards, and non-institutional environments like home births and community-based care. Together, they offered a broad, culturally grounded lens on how labour pain is perceived, experienced, and managed across different parts of the world.

The Heart of Labour: Sociocultural Forces Shaping Pain, Perception, and Care

Labour pain is a universal experience, yet its perception and expression vary significantly across cultures and contexts [20,21]. Religious and spiritual beliefs play a key role in shaping how women respond to labour pain [21]. One study found that a woman’s social environment, including support persons, caregivers, hospital staff, and even unfamiliar observers, shapes her interpretation of pain as either purposeful or threatening, influencing her decision to seek pain relief [22].

Comparative cross-cultural research reveals significant variation in how women express labour pain, shaped not only by religious affiliation but also by regional customs, healthcare settings, and degrees of acculturation. For instance, a Spanish study assessing 246 women with the Rating Scale of Pain Expression during Childbirth (ESVADOPA) scale found that Muslim women were the most vocally expressive, followed by Jewish, Atheist, and Christian women, suggesting that cultural-religious interpretations may legitimise or constrain vocal expressions of pain [23,24]. In this context, Muslim women were more likely to exhibit screaming or crying behaviours, perhaps normalised or even expected within their cultural setting [25]. However, other studies report opposite trends. For example, in a qualitative study in Jordan, Muslim women displayed stoicism, often suppressing pain to conform to ideals of maternal sacrifice and religious endurance [10]. Similarly, Pakistani Muslim women in the UK were found to underreport or internalise pain, influenced by both modesty norms and fear of medical judgment [8]. This intra-religious variability suggests that religious identity alone is insufficient to predict pain expression. Instead, intersections of religiosity, migration status, and institutional norms play a significant role. While Berber women in North Africa have been described as openly vocal and expressive during labour, Eastern European and sub-Saharan African women in European hospitals often remain silent, shaped by cultural ideals of strength and control, or by fear of racialised medical surveillance [26]. Moreover, clinical settings and language barriers may exacerbate silence among migrant women, not because of cultural stoicism alone, but due to institutional disempowerment [27]. These contrasting findings emphasise the need for comparative, context-sensitive ethnographic and clinical research that goes beyond essentializing religion or ethnicity. They highlight how labour pain expression is dynamically shaped by local norms, healthcare encounters, and power asymmetries, and call for a more nuanced understanding of maternal agency that is sensitive to both cultural variability and structural constraints.

In Ghana, a qualitative study found that women regarded crying during labour as a sign of weakness and instead endured pain silently, driven by sociocultural expectations [11]. The presence of a chosen birth companion was associated with improved pain coping and reduced vocal expression of pain - an approach also endorsed by the WHO in 2018 [28,29]. Language barriers further complicated women’s ability to communicate pain, often leading to misinterpretation by medical staff and reduced control over the birthing experience [30-33]. These communicative challenges highlight how sociolinguistic mismatches between patients and providers can exacerbate disparities in pain assessment and response. A study of Jewish and Bedouin parturients found that those assessed by ethnically dissimilar caregivers reported lower pain scores, reflecting interethnic bias in pain assessment [34]. Additional research identified racial bias in pain management, with women of colour perceived to have a higher pain tolerance than white women, resulting in disparities in analgesia administration [35]. Such biases stem from historically rooted stereotypes, reflecting how intersectionality between race and gender continues to influence clinical judgments in labour settings.

Educational background and cultural assimilation also influenced labour pain perception. For example, Middle Eastern women with lower educational attainment exhibited more pain behaviour compared to educated Western women [36]. In Nigeria, a study found that exposure to Western medical practices correlated with increased pain perception among 765 parturients [37]. In contrast, women in Northern Ireland reported that individual and environmental factors shaped their perception of pain, with a sense of personal control helping to alleviate discomfort [38]. A qualitative study in Ireland described labour as a personal journey, where pain was internalised and recalled as part of a transformative life event [39]. This interpretation frames labour pain not only as a physiological event but as a culturally constructed rite of passage, shaped by personal and collective memory. In some contexts, labour was described as “Gezellig” (Dutch for cozy), a perception shaped by supportive birthing partners, the availability of water births, and the choice of home birth [40]. Education appeared to influence how women differentiated labour pain from illness-related pain, though findings were mixed [41,42].

Sociocultural factors also impacted women's choices regarding childbirth settings. A study of 1,649 Nordic women who opted for home births found that autonomy, even in the face of intense pain, allowed for a more manageable labour experience [43]. However, some women sought early admission to hospitals due to anxiety and pain, despite midwives advising otherwise [44]. In rural Mozambique, proximity to clinics increased the likelihood of institutional deliveries, while seasonal and economic factors promoted home births [45]. In Ethiopia, institutional delivery rates remained low (12.1%), with many citing traditional norms, sudden labour onset, and family influence as reasons for home births [46]. These findings suggest that decisions around place of delivery are shaped by more than just logistics - they are deeply embedded in sociocultural narratives. For many women, giving birth at home is not merely a necessity but a continuation of traditional norms that valorise endurance, privacy, and autonomy. In contrast, institutional settings may be avoided not only due to infrastructural barriers but also due to perceived emotional neglect, loss of control, or fear of mistreatment. This interplay between cultural ideals of stoicism and systemic gaps in respectful, supportive care underscores the complexity of maternal health choices in diverse contexts.

In India, 22% of women deliver at home, with one-third perceiving institutional delivery as unnecessary. This was often linked to domestic violence, lack of autonomy, and socioeconomic disadvantage [47]. Additional studies identified nuclear family structures, late antenatal care (ANC) registration, absence from India's Janani Suraksha Yojana (JSY) program, and previous home births as contributing factors [42,48]. JSY is a conditional cash transfer program that incentivises institutional births, aiming to reduce maternal mortality. In contrast, Sweden promotes homebirths under certain conditions, while New Zealand emphasises lead maternity care, where a primary midwife provides continuity throughout the perinatal period. These policy contexts influence how women perceive control, choice, and support during labour [3,10-13]. This implies that governmental childbirth policies also shape women's experiences. In Zambia, although institutional births were preferred, women reported barriers including inadequate resources and disrespectful treatment [49]. Similarly, in Tanzania, most women delivered in health facilities due to improved socioeconomic conditions and ANC quality, though some ethnic groups still favoured home births [50].

Perception of pain was also influenced by antenatal education and caregiver support. A Nigerian study reported that 75.2% of women experienced severe labour pain, with higher social status and prior antenatal education linked to greater pain awareness [51]. A qualitative study involving 21 nulliparous women found that caregiver attitudes significantly influenced whether the childbirth experience was viewed positively or negatively [22]. Another study emphasised the roles of antenatal classes, labour induction, partner support, and emergency caesarean sections in shaping pain perception [52]. Focus Group Discussions among ten Nigerian mothers revealed that primiparous women perceived labour pain as more severe, with midwives’ instructions playing a positive role in pain interpretation [53]. In Helsinki, long labour durations, unplanned C-sections, and inadequate support were associated with negative childbirth experiences, shaped by the woman's subjective perception of pain [54]. These studies illustrate how deeply rooted cultural norms around childbirth, such as traditional home deliveries or stoic endurance of pain, continue to shape maternal choices across regions. However, these cultural continuities often intersect with systemic inadequacies, such as limited access to respectful care, poor infrastructure, and lack of pain management resources. When cultural preferences meet structural constraints, women may be left navigating childbirth in environments that neither fully respect tradition nor guarantee safe, dignified care. Addressing this duality requires culturally sensitive interventions that are simultaneously responsive to institutional shortcomings

Cross-cultural studies underscored the difficulty some women faced in adapting to Western models of childbirth. In Jordan, 92% of women anticipated a negative childbirth experience due to differences in pain management expectations [9,55]. Iranian studies highlighted how environmental stressors, such as crowded delivery rooms, immobility, and lack of fluids, heightened perceived pain during labour [56,57].

Taken together, these findings reinforce that labour pain is not just a physiological event, but a deeply personal and socially shaped experience. Women’s perceptions and expressions of pain are influenced by a complex interplay of cultural norms, religious beliefs, institutional settings, and broader social structures. Factors such as migration, language barriers, and policy environments often mediate how pain is expressed, understood, or silenced. While common threads emerge, like the role of stoicism, caregiver support, and the impact of marginalisation, there are also important contradictions that reflect the diversity of women's lives and circumstances. This highlights the need for culturally responsive, respectful maternity care that acknowledges both the uniqueness of each woman’s experience and the systemic realities in which childbirth takes place.

Perception Versus Reality: Exploring the Similarities and Differences Between Anticipated and Experienced Labour Pain

Studies examining women’s perceptions of labour pain reveal a complex interplay between expectations and actual experiences. A cross-sectional study involving 132 pregnant women in Nigeria, assessed using the Visual Analogue Scale (VAS), found that most participants rated labour pain as severe (mean score: 7.0), and 86.4% expressed a desire for pain relief [58]. A similar study among 221 postpartum women in Rivers State, Nigeria, observed a direct correlation between pre-labour pain expectations and actual pain perception during childbirth [59]. In Turkey, an observational study with 230 multiparous women demonstrated that those with lower expectations of pain reported lower levels of perceived pain, underscoring the influence of anticipation on pain experience [60].

Qualitative studies further highlight the psychological coping mechanisms employed by women during labour. In Australia, 19 women described shifting between two cognitive states - acceptance and aversion - to manage labour pain [19]. A Swedish study conducted at an alternative birth centre found that although women struggled with trust in themselves and their caregivers, many viewed labour pain as a natural phenomenon, encapsulated by one respondent's remark: “I think it's a happy pain, though it's hell” [58]. Another study challenged this notion, suggesting that women’s positive reflections on pain were more indicative of satisfaction with their coping strategies than with the pain itself [61]. Nonetheless, many women evaluated their painful experiences positively, as the pain was often eclipsed by the emotional fulfilment of childbirth [62].

These findings also reveal a lack of structured evaluative or preparatory processes prior to labour that might improve women's experiences. For instance, a cross-sectional study of 100 Nigerian parturients found that although many experienced severe labour pain, they considered it a “normal experience that every woman goes through in her life” [63]. Another Nigerian study showed that neither the presence of a companion, counselling, nor antenatal education significantly influenced pain perception, despite 73% of participants believing in their utility [64]. A separate study linked poor pain perception to tokophobia, the pathological fear of childbirth [65].

Expectations not only shaped immediate experiences but also had long-term implications. One study found an inverse relationship between positive expectations and perceived pain during labour, suggesting that overly optimistic views could lead to heightened distress [66]. In a randomised controlled trial, viewing foetal images during labour was shown to reduce anxiety and perceived pain, suggesting that visual cues may enhance coping capacity [67]. Likewise, a New Zealand study of 230 postpartum women found that stronger birth self-efficacy was associated with lower pain and emotional distress [68].

However, the accuracy of postpartum recall remains debatable. Two studies indicated that women’s recollections of labour pain were inconsistent with their immediate post-birth accounts, especially among primiparous women [69-71]. A comparative study of 141 primiparous and 99 multiparous women revealed that childbirth education significantly reduced perceived pain severity, particularly when physical factors such as height, weight, cervical dilation, and contraction intensity were accounted for [1].

Prolonged labour was often associated with heightened distress. A qualitative study noted that women undergoing long and difficult labours described feeling as though they were in life-threatening situations due to the unrelenting pain [71]. Conversely, other studies argued that women often distinguish labour pain from pathological pain, viewing it as a purposeful part of childbirth rather than a condition requiring intervention [3,41].

However, antenatal fear and negative expectations have consistently been linked to poorer labour outcomes. An Australian study noted that women who approached labour with fear were more likely to report negative experiences [72]. Across various studies, women were found to anticipate intense, often unbearable pain [38,73,74]. One study suggested that women who expected labour to be “quite painful” had more realistic expectations and subsequently more positive experiences [75]. Perception clearly influenced outcome: women who experienced more pain than expected often reported feelings of failure, while those whose expectations aligned with reality were more likely to feel empowered [39,76]. However, at least one study reported no significant difference between expected and experienced pain levels [70]. Notably, underestimation of pain was commonly associated with a lack of preparedness, which often translated into more negative birthing experiences [38,39,73-79].

These findings underscore the powerful role of expectations in shaping how women perceive and remember labour pain. While some women viewed pain as purposeful or even transformative, others experienced it as overwhelming and traumatic-particularly when their expectations did not align with reality. Across contexts, it is evident that psychological preparedness, antenatal education, and a supportive environment can meaningfully influence pain perception and coping. At the same time, the variation in experiences-ranging from acceptance to fear, from resilience to distress-highlights the deeply personal and subjective nature of labour pain. Acknowledging this complexity is essential to improving care, as mismatched expectations, inadequate preparation, and emotional distress can compromise both immediate and long-term birthing experiences. Ultimately, bridging the gap between perception and experience will require more than education; it calls for empathetic, individualised support that prepares women not just for pain, but for the wide range of emotions that labour may evoke.

Perceptions and Experiences: Key Drivers in Shaping Labour Pain Management Approaches

Women’s choices regarding pain management during labour were closely linked to their anticipation of pain [72]. Numerous studies have shown that women generally desired access to effective pain relief during childbirth [38,59,71,80-83]. In a study involving 486 multiparous Yoruba women in South-Western Nigeria, 14% expressed anxiety about labour pain and 33% reported a desire for its complete elimination. Despite widespread acknowledgement of labour as painful, many women tolerated it due to limited awareness or access to pain relief methods, highlighting a stark contrast with women’s experiences in more resource-rich settings. Another study among 281 pregnant Yoruba women found that 86% wished to receive pain relief, despite cultural norms suggesting that it was unnecessary. By contrast, a cross-sectional study among 82 Igbo women found a tendency to endure labour pain without requesting relief, further pointing to cultural expectations surrounding pain tolerance [71]. In Enugu, a study revealed insufficient provision of obstetric analgesia in health facilities [64], and among 212 Nigerian parturients, 82% lacked knowledge of available pain relief methods [72]. Similarly, in rural Kenya, 389 women rated their labour pain as severe to unbearable, yet only 43% received any form of analgesia [74].

In England, a large prospective study revealed that although most women expected labour to be intensely painful, many were hesitant to use pharmacological pain relief due to concerns about drug exposure, which contributed to heightened anxiety and negative birth experiences [73]. In Nigeria, 77.5% of women rated their labour pain as very severe, and nearly 96% indicated a desire for pain relief in future deliveries. A narrative inquiry involving five low-risk Ghanaian women illustrated that while they considered labour pain to be intense, they endured it due to inadequate pain management options and a lack of emotional and physical support during childbirth [80].

While many women expressed a desire for pain relief, others hoped to manage labour pain with minimal or no pharmacological intervention. Studies have shown that some women preferred non-pharmacological strategies or limited drug use, often citing a desire for a more 'natural' birthing experience [38,39]. In the United States, a national survey of 1,382 women reported that 75.3% used epidural analgesia, 25% used narcotics, and 1.5% used nitrous oxide. Notably, 70% also employed non-pharmacological techniques, such as doula support, breathing exercises, birth balls, and position changes [75]. A similar trend was observed in Poland, where epidural analgesia was considered the gold standard, but water births yielded the highest satisfaction rates, with 95% of parturients reporting a positive experience [76].

Importantly, one study found that when women were educated about labour and had greater autonomy in decision-making, they were more likely to request timely epidural analgesia, leading to improved pain management outcomes [77]. A retrospective cohort study in Singapore involving 10,170 parturients assessed satisfaction following epidural use: 31.8% were not satisfied, 32.2% were very satisfied, and 35.9% reported being generally satisfied [78].

However, disparities in access to and knowledge of pain relief methods persisted. A qualitative study involving immigrant women speaking Albanian, Arabic, Farsi, Tamil, or Tigrigna revealed limited understanding of epidural analgesia, which created conflicting perceptions that influenced their pain management choices [79]. Recent innovations, such as the use of virtual reality (VR) during labour, have also demonstrated potential in reducing pain perception. A qualitative study from the Netherlands showed that VR use contributed positively to labour experiences [80]. Other non-pharmacological methods, including guided breathing and nurse-administered massages, were also reported to effectively reduce pain intensity and enhance maternal satisfaction [81-83]. Some women opted for a hybrid approach, combining pharmacological methods with inhaled agents like Entonox, which they perceived as more natural and less intrusive than injectable drugs [74].

Across diverse settings and cultural contexts, the overarching finding remains consistent: most women desire accessible and effective pain relief during labour. Even those initially opposed to pharmacological interventions often reconsidered their stance after encountering severe labour pain, highlighting the importance of comprehensive antenatal education and flexible, woman-centred pain management strategies.

These studies reveal that pain management choices during labour are shaped by a complex interplay of cultural expectations, personal beliefs, systemic access, and informed autonomy. While many women expressed a desire for effective pain relief, their ability to access and choose suitable methods was often constrained by gaps in knowledge, limited availability, or fears related to medical interventions. Even in higher-resource settings, concerns about safety and a preference for natural childbirth shaped women's decisions. What emerges is not a simple preference for or against pharmacological options, but a broader need for informed choice, respectful support, and meaningful education. Whether women opt for epidurals, breathing techniques, or hybrid approaches, the common thread is their desire to feel prepared, heard, and in control. These findings underscore the importance of pain management strategies that are flexible, culturally sensitive, and centred on the individual needs and expectations of labouring women.

Strengths and Limitations

This review comprehensively addresses sociocultural factors shaping women’s perceptions and experiences of labour pain, incorporating diverse cultural contexts, religious beliefs, and socioeconomic conditions. Following PRISMA-ScR guidelines, the study includes various study types, namely, RCTs, cohort studies, case reports, and qualitative studies, ensuring a well-rounded approach. The focus on cultural sensitivity highlights the importance of tailoring pain management strategies to diverse populations. With global perspectives from regions like Nigeria, Ghana, Sweden, New Zealand, and India, the findings offer practical implications for improving antenatal care and pain management strategies. However, the review has limitations, including the exclusion of non-English studies, potentially miss valuable insights from diverse cultural contexts. The study selection process may have introduced bias, and the variation in study designs complicates comparisons. This review does not explore the long-term effects of labour pain or postnatal experiences. In addition, we have not taken into account the quality appraisal or risk of bias assessment, as this is a scoping review.

Perspective

By identifying key determinants such as religious beliefs, ethnicity, language barriers, and socioeconomic status, the findings underscore the need for a culturally responsive framework in maternal care. To operationalise this, each factor can be mapped onto specific intervention strategies. For example, religious beliefs can inform the design of faith-sensitive antenatal education modules, while language barriers can be addressed through multilingual doulas or culturally competent interpreters. Ethnic and socioeconomic disparities could guide targeted outreach and subsidised access to pain relief options. This framework would enable clinicians and policymakers to move from general awareness to tailored implementation. Policies should therefore focus on integrating cultural competency training into medical curricula, developing informed consent protocols that reflect diverse values around childbirth, and investing in community-based education to enhance maternal autonomy and preparedness. Such a structured approach would not only promote equitable access to pain management but also ensure that women’s expectations are aligned with culturally respectful and evidence-based care.

## Conclusions

This review identified three key themes: (1) the influence of sociocultural factors on labour pain perception and management, (2) the perception and experience of labour pain, and (3) the perception and experience of pain management. Women’s antenatal perceptions of labour pain, often shaped by socio-familial norms and unpreparedness, determined whether their experience was positive or negative. While negative perceptions of pain management often shifted post-experience, higher pain intensity increased the desire for pain relief options. Sociocultural factors like religious beliefs, ethnicity, language barriers, socioeconomic status, antenatal education, and the presence of birth companions significantly influenced these perceptions. Lack of preparation and awareness about pain relief contributed to inconsistencies between expectations and reality. Antenatal education can bridge this gap by strengthening realistic expectations and informed decision-making. Ultimately, sociocultural dynamics shape how women perceive and experience labour pain, highlighting the need for culturally sensitive care.

To translate these insights into practice, several actionable steps can be considered: i) Integrate culturally tailored antenatal education programs that address labour pain expectations, pain relief options, and coping strategies. ii) Train healthcare providers in culturally competent care, especially in recognising and responding to diverse expressions of pain. iii) Improve communication pathways, particularly in multilingual settings, to ensure women’s pain is effectively understood and addressed. iv) Enhance access to both pharmacological and non-pharmacological pain relief, especially in under-resourced settings. v) Encourage the inclusion of birth companions to support emotional well-being and bridge cultural expectations during labour. These strategies could help reconcile sociocultural expectations with systemic realities, ultimately promoting more respectful and responsive maternity care.
